# Blending in or standing out? The disclosure dilemma of ad cues of social media native advertising

**DOI:** 10.3389/fpsyg.2025.1636910

**Published:** 2025-08-13

**Authors:** Maike Hübner, Julia Thalmann, Jörg Henseler

**Affiliations:** ^1^Usability and Interaction Laboratory, Department of Business Administration and Economics, Ruhr West University of Applied Sciences, Mülheim, Germany; ^2^Department of Design, Production and Management, University of Twente, Enschede, Netherlands; ^3^Nova Information Management School, Universidade Nova de Lisboa, Lisbon, Portugal

**Keywords:** native advertising, disclosure, visual attention, ad blindness, sponsorship, advertising effectiveness

## Abstract

**Introduction:**

As social media platforms increasingly rely on native advertising embedded within user feeds, an open question is whether sponsored posts garner comparable, greater, or reduced attention relative to surrounding non-sponsored content. Subtle cues (e.g., disclosures, call-to-action (CTA) buttons) may alert users to the commercial nature of these posts or remain unnoticed in rapid-scroll environments. In this study, we aimed to disentangle the dynamics of the “disclosure dilemma” in native advertising, examining whether ad cues blend into the content flow unnoticed or stand out and prompt rapid disengagement.

**Methods:**

This study adopted a mixed-methods design with 152 participants randomly assigned to one of three mock-up Instagram feeds containing both sponsored and organic posts. Eye-tracking measured visual attention (dwell time, fixation counts), while cued-retrospective think-aloud (CRTA) interviews probed underlying user rationales for focusing on or overlooking specific content.

**Results:**

Sponsored posts received significantly less dwell time and fewer fixations than their organic counterparts, indicating persistent ad avoidance despite a native design. Moreover, early fixation on disclosures or CTA elements in sponsored posts often triggered an immediate decline in further engagement, functioning as “flags” that activate learned avoidance. CRTA data revealed divergent user interpretations: some participants felt misled by the subtlety of sponsored posts, whereas others remained unaware of disclosures until late in their viewing.

**Discussion:**

These findings point to an evolving form of “native ad blindness,” shaped by rapid heuristic scanning and schema-based recognition of minimal ad signals. Conceptually, this study refines theories of banner blindness, persuasion knowledge, and dual-process filtering, providing practical insights for advertisers seeking to balance transparency with user engagement amid competition from non-sponsored content.

## 1 Introduction

Over the past decade, social media platforms have become crucial channels for brand communications. In particular, Instagram and other feed-based applications have shifted away from easily identifiable banner ads toward native advertising, wherein sponsored content is interspersed among organic content with minimal distinction (Einstein, 2015; Marks et al., 2019; Matteo and Zotto, 2015). The purported advantage of native advertising lies in its capacity to appear “natural,” potentially circumventing ad avoidance that plagues more traditional web banners (Apostol, 2020; Asquith and Fraser, 2020; Chung and Kim, 2021; Jiang et al., 2017). However, this same seamlessness raises legitimate concerns over transparency (Jung and Heo, 2019; Lee et al., 2016; Pasandaran and Mutmainnah, 2020; Wojdynski and Evans, 2016): Can users consistently discern between authentic organic content and sponsored content when ads are deliberately camouflaged?

From a regulatory standpoint, agencies such as the Federal Trade Commission (FTC) in the United States have emphasized the necessity of clear and conspicuous disclosures so that users can understand when a post is commercial (Federal Trade Commission, 2013). However, these disclosures often appear as subtle “sponsored” labels, rendering them less visually striking compared with classic banner ads. Consequently, research has shown that consumers still fail to classify these disclosed posts as advertising (Boerman et al., 2017; Pasandaran and Mutmainnah, 2020; Wojdynski and Evans, 2016). The current study further questions the reasons for this and aims to discern whether these micro-cues are seen or overlooked, and whether they function as an “ad alert,” influencing users’ behavior (reaction) to it.

Recent findings point to an extension of the banner blindness phenomenon to native ads (Maslowska et al., 2021). Banner blindness, a term coined by Benway (1998), originally referred to a phenomenon in which users learn to ignore specific page regions or design templates that are widely associated with advertising. Early empirical work found that even stimuli placed in close proximity to editorial content were missed if they resembled the despised “banner” format (Benway, 1998). As websites evolved, so did user avoidance mechanisms: people increasingly developed scanning strategies to swiftly skip anything that resembled a banner (Cho, 2004). However, native advertising in a scrollable feed complicates this paradigm. Recent studies (e.g., Maslowska et al., 2021) provide preliminary evidence that once users detect these tiny disclosures, they mentally categorize the post as “commercial” and may disengage. In other words, banner blindness may still manifest—but in the new guise of skipping any feed post that exhibits minimal “ad flags.” This notion of native ad blindness suggests that even the slightest hint of persuasion can trigger learned avoidance. Yet, not all users skip ads immediately (Kim, 2017; Noguti and Waller, 2020). Some might pause if the content looks highly relevant (Chung and Kim, 2021) or if the disclosures are overlooked.

A growing body of research has explored how consumers visually and cognitively engage with native advertising (NA), yet much of this work examines advertising stimuli in isolation. Prior studies investigated how disclosure formats (Binford et al., 2021; Boerman and Müller, 2022; Klein et al., 2022), ad placement (De Keyzer et al., 2023; Wang and Hung, 2019), and friend referrals (Maslowska et al., 2021; Windels et al., 2018) affect the visual attention to ads in social media feeds. For example, in-feed formats capture more fixations than banner ads (De Keyzer et al., 2023), and social-proof cues (in the form of friend referrals) boost early attention (Windels et al., 2018); yet, explicit “#ad” labels often go unread, a phenomenon termed “invisible transparency.” (Binford et al., 2021). However, these studies present ads as decontextualized stimuli rather than stimuli embedded in a continuous, live-style scrolling feed alongside organic posts. Even those that have focused on dynamic display (e.g., Maslowska et al., 2021; Windels et al., 2018) have measured sponsored-post fixations in isolation, without benchmarking them against adjacent organic content. However, unlike traditional digital formats, native ads are deliberately designed to visually blend into their environment, minimizing the perceptual boundaries between commercial and organic content. This blending, while often effective in reducing resistance at the surface level, introduces a deeper dilemma: whether such ads remain unnoticed altogether or whether they trigger disengagement as soon as their persuasive intent is revealed. Recent studies have begun to address parts of this broader landscape but remain siloed. Some explore NA in non-social media contexts such as digital journalism (e.g., Zamith et al., 2021), while others examine user attitudes toward NA on platforms like Twitter and Instagram through survey methods (e.g., Kim et al., 2021; Wang, 2025), without capturing real-time attentional dynamics. Visual attention research, meanwhile, has focused on Facebook (e.g., García-Carrión et al., 2025; Lee et al., 2024) or on non-social media formats like banner ads or text passages (e.g., Luke and Jensen, 2023; Ronconi et al., 2025). Although a few studies have applied eye-tracking to NA on social media (e.g., Ding et al., 2022), they emphasize stylistic framing (e.g., metaphorical vs. literal language) rather than the recognition and behavioral consequences of disclosure cues.

Consequently, the literature lacks an integrative study that triangulates native advertising, social media, and visual attention. In particular, there is no existing work that benchmarks gaze behavior on sponsored posts relative to organic content within an ecologically valid, mobile-first feed. Beyond this broader absence, the field also lacks a granular understanding of how visual attention is allocated within the sub-areas of a post (content, branding elements, disclosure, and social cues) and how these micro-level patterns resolve the core “disclosure dilemma” of blending in (evading detection and sustaining engagement) versus standing out (prompting immediate dismissal). To address these empirical and theoretical gaps, this study answers three interrelated research questions (RQs) that build on one another:

*RQ1* (overall attention): In a realistic mobile-first feed, how does total gaze time on sponsored posts differ from that on adjacent organic posts?

*RQ2* (component attention): Within sponsored posts, how is visual attention apportioned among the four core subareas (content, branding elements, disclosure, and call-to-action (CTA) buttons) when competing directly with organic content?

*RQ3* (ad-flag dynamics): How do early fixations on disclosure labels or CTA buttons influence subsequent engagement with the rest of the post, thus leading to the “blending in” versus “standing out” dilemma?

These questions reflect an escalating analytical lens, from overall attention differences at the post level (RQ1), to within-post fixation patterns (RQ2), to the behavioral consequences of encountering specific ad-related cues (RQ3). Collectively, they aim to disentangle the dynamics of the “disclosure dilemma” in native advertising: whether ad cues blend into the content flow unnoticed or stand out and prompt rapid disengagement.

To answer these questions, this study adopted a mixed-methods approach in a simulated, mobile-first social media environment that mirrors real-world Instagram use. Participants scrolled through a balanced mix of sponsored and organic posts, during which their gaze behavior was tracked using eye-tracking technology. Subsequently, participants engaged in cued-retrospective think-aloud interviews, allowing us to interpret not just where they looked but why. Together, the findings demonstrate that while native ads can partially “blend in,” even minimal ad flags, when actually fixated, trigger rapid dismissal. Conversely, undiscovered cues allow ads to slip under users’ radar and enjoy engagement levels comparable to those of friend posts. These results highlight the delicate balance advertisers must strike between ethical transparency and maintaining user attention.

## 2 Materials and methods

### 2.1 Theoretical background and methodological approach

To address the disclosure dilemma of “blending in” versus “standing out” in real browsing, we move beyond isolated feature tests. We developed a theoretically grounded, three-stage framework to understand visual attention to native advertising. We integrated five interlocking theoretical perspectives: schema theory, dual-process theories (DPTs), the persuasion knowledge model (PKM), the limited capacity model of mediated message processing (LC4MP), and the eye-mind hypothesis. Rather than applying these models in isolation, they were mapped directly onto the empirical architecture of this study, allowing each to illuminate a distinct layer of the ad attention process, from broad attention allocation patterns, to within-post gaze distribution, to the temporal dynamics of recognition and avoidance.

In high-velocity, low-involvement environments such as Instagram, users typically engage in rapid scrolling punctuated by momentary fixations. Under such conditions, attention is highly selective, and users rely heavily on heuristics. DPTs of information processing, including the heuristic–systematic model (Chaiken et al., 1989), elaboration likelihood model (Petty and Cacioppo, 1986), and Kahneman’s (2011) System 1/System 2 framework, converge on the notion that people process incoming stimuli through two qualitatively distinct systems. In contexts where motivation and cognitive capacity are limited, such as passive social media use, users’ default to System 1, an intuitive and fast-acting mode that enables quick judgments based on surface cues. In the context of native advertising, this implies that even minimal cues, such as a disclosure, CTA button, or the absence of typical social features, can trigger a heuristic classification of the post as commercial. Such cue-driven shortcuts reduce the likelihood of deeper elaboration (System 2) and often lead to post-level disengagement. However, whether this effect holds across a full, scrollable feed populated with competing organic content remains unclear. To test this, our first research hypothesis addresses attention at the post level:

*H_1_* (post-level hypothesis): Users allocate less visual attention to sponsored posts than to organic posts in mobile-first social media environments.

However, native advertisements do not consist of a monolithic block of content; rather, they are composed of interdependent sub-elements, each carrying distinct functional and persuasive value. Once a user pauses their scroll and visually engages with a post, attention is distributed across these internal components: the main content image, the source or profile name, engagement metrics, disclosure labels, and CTA prompts. At this finer level of analysis, the PKM (Friestad and Wright, 1994) becomes particularly relevant. The PKM holds that once individuals recognize a message as advertising, they do not merely lose interest, they actively deploy cognitive resistance strategies, including skepticism, attributional discounting, or strategic avoidance. In a native ad, such strategic coping may take the form of reducing attention to branding elements, skipping over CTA prompts, or ignoring disclosure labels altogether. Importantly, the PKM implies that attention is not only suppressed globally but also selectively redistributed away from the persuasive elements of a message once the user detects its commercial intent.

Simultaneously, schema theory (Axelrod, 1973; Bartlett, 1932) offers an explanatory model for the rapidity of such recognition. Through accumulated exposure, users develop mental templates—also referred to as schemata—of what advertising typically looks like, even in its most camouflaged forms. When a particular visual configuration (e.g., a “sponsored” label near a profile name, a high-contrast CTA button) matches this schema, recognition can occur almost instantaneously, often without conscious deliberation. In this context, schematic processing and the PKM jointly predict a reallocation of attention away from ad-like components. However, while past studies have tested isolated design features (e.g., disclosure format, brand prominence) (Binford et al., 2021; Boerman and Müller, 2022), research has yet to examine how users distribute attention across native ad components within a live-scroll environment and how this differs from organic content; this leads us to our second hypothesis:

*H_2_* (component-level hypothesis): Within sponsored posts, attention is unequally distributed across components, with advertising-related elements (e.g., disclosures, CTA buttons) receiving less attention than content-oriented elements (e.g., imagery or social cues).

Crucially, visual attention is not only spatially differentiated across post elements but also unfolds temporally. When and in what order users fixate on different post elements have significant consequences for how the post is ultimately processed. For instance, if a disclosure label or CTA button is among the first elements to be fixated upon, this early recognition of persuasive intent may further suppress cognitive engagement with the rest of the post. To understand this mechanism, we draw on two final perspectives: the LC4MP (Lang, 2017) and the eye-mind hypothesis (Just and Carpenter, 1980).

The LC4MP posits that individuals have a finite pool of cognitive resources to allocate to media processing. In high-load environments, such as scrollable social feeds, only a fraction of the available content can be fully processed. When a user’s attentional resources are depleted or when a cue is not sufficiently salient, it may simply not be processed. This can result in advertising elements being present but functionally invisible. Conversely, when a disclosure or CTA is noticed early, it may monopolize limited resources or trigger motivational resistance via PKM, resulting in immediate disengagement from the rest of the post. These effects are best understood in light of the eye-mind hypothesis, which asserts a near-synchronous relationship between where users look and what they are cognitively processing (Just and Carpenter, 1980). Accordingly, if a user’s first fixation lands on a disclosure and is followed by reduced attention to other components, this temporal sequence can be interpreted as an ad recognition event leading to strategic disengagement. Prior research has often treated ad recognition as binary (noticed versus unnoticed), whereas our approach captures its temporal dynamics. We ask not only whether a cue was seen, but also when and what happened next, informing our third hypothesis:

*H_3_* (temporal-dynamic hypothesis): Early attention to advertising cues predicts reduced engagement with other post components, consistent with schema-based recognition and strategic avoidance.

Together, these theoretical lenses underscore the complex and layered nature of visual attention in the context of native advertising. From initial perceptual salience (LC4MP), through schema-based ad recognition and PKM-driven avoidance, to heuristic and systematic processing modes (DPTs), these models predict nuanced, temporally sensitive responses to subtle ad cues. The research hypotheses offer a multi-layered model of visual attention to native advertising. H_1_ addresses post-level engagement differences, testing whether native ads attract less attention overall in dynamic, mobile-first feeds. H_2_ moves to the component level, exploring how attention is differentially allocated within a post once it has been visually engaged with. H_3_ addresses the sequential flow of recognition and avoidance, capturing the cognitive consequences of early cue detection. Each hypothesis reflects a distinct facet of the disclosure dilemma: how native ads navigate, or fail to navigate, the boundary between blending in and standing out, as well as the role of the disclosure within this. As theory alone cannot resolve the disclosure paradox, we require real-time evidence of what users see, in what sequence, and how that gaze translates into recognition and behavioral outcomes. To empirically test these dynamics, this study explored the three research hypotheses across three stages, moving from post-level attention (Stage 1) to component-level processing (Stage 2), and finally to temporal-sequential recognition (Stage 3a) as well as the reasoning of attention allocation (Stage 3b). Table 1 presents a full mapping of the broader theoretical hypotheses onto specific operational hypotheses, areas of interest (AOIs), and metrics. The following sections outline how these research hypotheses were operationalized in a multi-method empirical study. Specifically, we implemented a three-stage mixed-methods design using mobile eye-tracking and cued-retrospective think-aloud (CRTA) methods to test the hypotheses articulated above.

**Table 1:** Operationalization of theoretical constructs and hypotheses across stages 1–3: metrics, AOIs, and expected outcomes.

	AOI	Post element	Metric	Hypothesized direction	Theoretical rational	Hypothesis
Stage 1	AOI 7	Overall post	Dwell time	↓ Sponsored < Organic	Banner blindness and schema theory predict users will disengage more quickly from sponsored content once minimal cues are detected. Dual-process models suggest this may occur through heuristic System 1 processing.	H₁ₐ: μ Dwell_Sponsored < μ Dwell_Organic
			Fixation count	↓ Sponsored < Organic	Sponsored posts may trigger reduced visual scanning due to learned avoidance of persuasive content (PKM, Eye-Mind Hypothesis).	H1b: μ Fix_Sponsored < μFix_Organic
Stage 2	AOI 1	Content image	Dwell time	↑ Organic > Sponsored	System 2 may be activated when evaluating content relevance; organic posts more likely to sustain deliberate engagement Kahneman (2011).	H2a: μ_Dwell_Sponsored^AOI1 < μ_Dwell_Organic^AOI1
			Fixation count	↑ Organic > Sponsored	Organic content may prompt exploratory scanning due to perceived authenticity and lower ad salience.	H2b: μ_Fix_Sponsored^AOI1 < μ_Fix_Organic^AOI1
	AOI 2	Engagement buttons (Like, Share, Comment Icons)	Dwell time	↑ Organic > Sponsored	Users interpret engagement features as socially driven cues; ad skepticism reduces processing in sponsored posts (PKM).	H2c: μ_Dwell_Sponsored^AOI2 < μ_Dwell_Organic^AOI2
			Fixation count	↑ Organic > Sponsored	Familiarity with icons may trigger habitual scanning (System 1); more active checking expected in organic posts (schema-based).	H2d: μ_Fix_Sponsored^AOI2 < μ_Fix_Organic^AOI2
	AOI 3	Source (Profile Picture and Name)	Dwell time	↑ Organic > Sponsored	Users more likely to evaluate source credibility for organic posts (System 2); less need to assess credibility in ads.	H2e: μ_Dwell_Sponsored^AOI3 < μ_Dwell_Organic^AOI3
			Fixation count	↑ Organic > Sponsored	Users may quickly check the source identity more often when not expecting promotional content.	H2f: μ_Fix_Sponsored^AOI3 < μ_Fix_Organic^AOI3
	AOI 4	Social Cues (Likes and Comments)	Dwell time	↑ Organic > Sponsored	Cialdinis Social Proof Theory: users consider peer metrics more informative in organic content. Sponsored content may trigger cognitive disengagement (PKM).	H2g: μ_Dwell_Sponsored^AOI4 < μ_Dwell_Organic^AOI4
			Fixation count	↑ Organic > Sponsored	Frequent but brief scanning aligns with System 1 processing; users habitually glance at social cues in organic content to assess relevance.	H2h: μ_Fix_Sponsored^AOI4 < μ_Fix_Organic^AOI4
Stage 3a	AOI 5_First	CTA button first fixation	Dwell time on AOI (1-4)*	↓ Early CTA > Shorter Dwell	CTA buttons act as visually salient, affordance-based cues that may trigger automatic recognition of persuasive intent (Norman, 2013; PKM).	H3a: μ_Dwell_EarlyFixAOI5 < μ_Dwell_NoEarlyFixAOI5
	AOI 6_First	Disclosure first fixation	Dwell time on AOI (1-4)*	↓ Early CTA > Shorter Dwell	Disclosures activate persuasion knowledge via top-down processing, leading to faster disengagement (PKM; Schema Theory).	H3b: μ_Dwell_EarlyFixAOI6 < μ_Dwell_NoEarlyFixAOI6
	AOI 6_AOI 5	Disclosure followed by CTA	Dwell time on AOI (1-4)*	↓ Sequential Fixation > Shorter Dwell	Sequential ad cue detection may reinforce persuasion knowledge, prompting disengagement if both cues are noticed early.	H13c: μ_Dwell_AOI6_AOI5 < μ_Dwell_NoAOI6_AOI5
	AOI 5_AOI 6	CTA followed by disclosure	Dwell time on AOI (1-4)*	↓ Sequential Fixation > Shorter Dwell	Automatic recognition of CTA followed by confirmation through disclosure may expedite disengagement.	H13d: μ_Dwell_AOI5_AOI6 < μ_Dwell_NoAOI5_AOI6

*Note Hypotheses H3a–d represent general patterns. To operationalize them, each fixation condition (AOI5_First, AOI6_First, AOI5_AOI6, AOI6_AOI5) was tested as predictors of dwell time across all attention targets (AOI1–AOI4) and across all sponsored posts (*N* = 31), resulting in 124 separate hypothesis tests. Therefore, we list generalized hypotheses (H13a–d) for Stage 3a, these are representative, not exhaustive. Each hypothesis condition (e.g., AOI5_First) was tested across multiple AOIs and posts.

### 2.2 Research design

This study adopted a mixed-methods approach, combining eye-tracking and CRTA methodologies to investigate user interactions with in-feed native advertising. The integration of quantitative (eye-tracking metrics) and qualitative (CRTA) data provides a multidimensional perspective on attention allocation (Bojko, 2013). Specifically, eye-tracking was chosen for its objectivity in capturing real-time attention (Scott and Hand, 2016). Building on Ehmke and Wilson’s (2007) work, the following primary metrics were recorded: fixation count (= number of distinct fixations on a specific AOI), dwell time (ms) (= total duration of gaze within an AOI), and time to first fixations (TTFFs; the sequence in which AOIs were viewed; e.g., whether disclosures were noticed before the main content). These metrics provide granular insights into whether participants visually engaged with sponsored content and how that engagement compares with the engagement with organic posts. High fixation counts or longer dwell times typically indicate deeper attention (Bojko, 2013; Holmqvist et al., 2012; Just and Carpenter, 1980). CRTA augments quantitative gaze data by probing the “why” behind gaze patterns. Showing participants their eye-tracking replay stimulates recall more effectively than a standard retrospective think-aloud (Bruun et al., 2021) method. The responses were audio-recorded and transcribed. Thematic content analysis was then performed using a codebook (see Supplementary Table 1) developed to categorize user comments on attention triggers, perceived ad recognition, and motivations for further or halted attention. Combining deductive and inductive coding enhances data reliability and depth (Bihu, 2024). The CRTA data triangulate with eye-tracking metrics. For example, if participants fixated briefly on a sponsored disclosure but then moved on, the CRTA transcripts helped clarify whether that reaction stemmed from ad avoidance, confusion, or another factor.

### 2.3 Experimental setup

A within-subjects design was selected to compare user attention toward sponsored (*n* = 8) and organic (*n* = 21) posts within a single mock-up Instagram feed (29 posts in total). This structure minimizes inter-individual variability and allows direct observation of how the same participant allocates attention to both sponsored and non-sponsored content. By controlling the feed layout (all posts shared uniform dimensions) and randomly assigning a post order, systematic bias was prevented (Miller, 2005; Rogers and Révész, 2019), thus ensuring internal validity. External validity was enhanced via the dynamic, scrollable feed that approximates real Instagram browsing. Participants navigated the feed autonomously, with a maximum scrolling time of 1 min 50 s. While Instagram session durations average approximately 2 min 44 s (Soax, 2024), actual attention to individual ads is far more fleeting. Industry findings show that the average ad receives less than 2.2 s of visual engagement on mobile (Neurons, 2025). Moreover, WARC (2022) highlights that brands have approximately 1.7 s to capture users’ attention before they decide to scroll on. Our time cap was therefore designed to emulate real-world conditions, acknowledging the rapid, low-effort evaluation processes that users typically engage in while browsing social media feeds. We opted against a fully forced-exposure paradigm (e.g., presenting one static post for a fixed duration) to capture more natural user interactions (De Keyzer et al., 2023; Windels et al., 2018). However, some constraints (maximum time limit) were imposed to maintain consistency across participants and approximate typical “fast-scrolling” behaviors online.

### 2.4 Stimulus material and presentation

The mock-up Instagram feed encompassed 29 posts (8 sponsored, 21 organic). The stimuli (posts) used in this study consisted of real advertisements that were actively running on social media platforms during the time of data collection. This decision was made to ensure ecological validity, allowing for a more accurate representation of how users interact with sponsored content in their everyday scrolling behavior. The selected ads were chosen based on a structured selection process to ensure diversity in industry, product category, and engagement type. The selection criteria were as follows: (1) advertisements from verified brand accounts and (2) relevance to popular consumer categories.

To simulate a broad user experience, posts spanned the most common Instagram themes, such as fashion, travel, food, media, music, and fitness, among others (Heepsy, 2023). After collecting a larger pool of active advertisements, the research team conducted an internal review and selected a subset of ads that best aligned with the study objectives, ensuring a balanced representation. Following prior industry reports (WhistleOut, 2022), the ratio of ads to organic posts (8:21) aimed to reflect a realistic (though somewhat ad-heavy) social feed without overwhelming participants. Ads were interspersed at random intervals, thus preventing participants from anticipating ad placements. Importantly, as this study focused exclusively on Instagram, this ratio aligns with prior findings indicating that Instagram’s ad density averages 20.6%, but some users experience peak ad saturation levels as high as 42% (WhistleOut, 2022). The ad ratio chosen in this study accounted for this variability in exposure, ensuring that participants encountered a level of ad saturation representative of both typical and high-exposure Instagram environments. To complement the sponsored content, organic posts were collected from real existing posts shared by the researchers’ families and friends. These posts were carefully curated to reflect typical user-generated content found on Instagram across various content categories. To prevent familiarity bias among participants, all identifying information (e.g., profile names, usernames, and any references to individuals or locations) was altered. Additionally, careful screening ensured that none of the organic posts featured content that could be personally recognizable or relatable to the study participants. This approach helped maintain the authenticity of the organic posts while eliminating potential confounding effects related to prior user familiarity. The final stimuli set was balanced to create a realistic yet experimentally controlled mock-up Instagram feed that mirrored the diversity and engagement levels of a typical user experience. This careful selection process allowed for a robust investigation of how users recognize, engage with, and respond to native advertising within their everyday media environment.

### 2.5 Participant recruitment and inclusion process

A total of 186 participants were recruited via convenience and snowball sampling from a university community and its surrounding area through multiple outreach channels, ensuring a diverse sample while maintaining accessibility for in-lab data collection. Recruitment efforts included announcements on the university website, social media outreach, and an online booking system that allowed participants to schedule their study sessions at their convenience. Flyers and posters were distributed in key locations to maximize participation. The inclusion criteria were as follows: participants had to be active Instagram users (using it ≥1 per day) to ensure familiarity with platform conventions; they had to have normal or corrected-to-normal vision (contact lenses allowed; eyeglasses not permitted due to eye-tracker compatibility issues) and not exhibit eye alignment abnormalities (e.g., strabismus, nystagmus) that could compromise gaze data accuracy. These recruitment criteria helped ensure internal validity and a sample representative of active social media users while controlling for data quality constraints and maintaining eye-tracking data reliability (Holmqvist et al., 2012).

**Figure 1:**
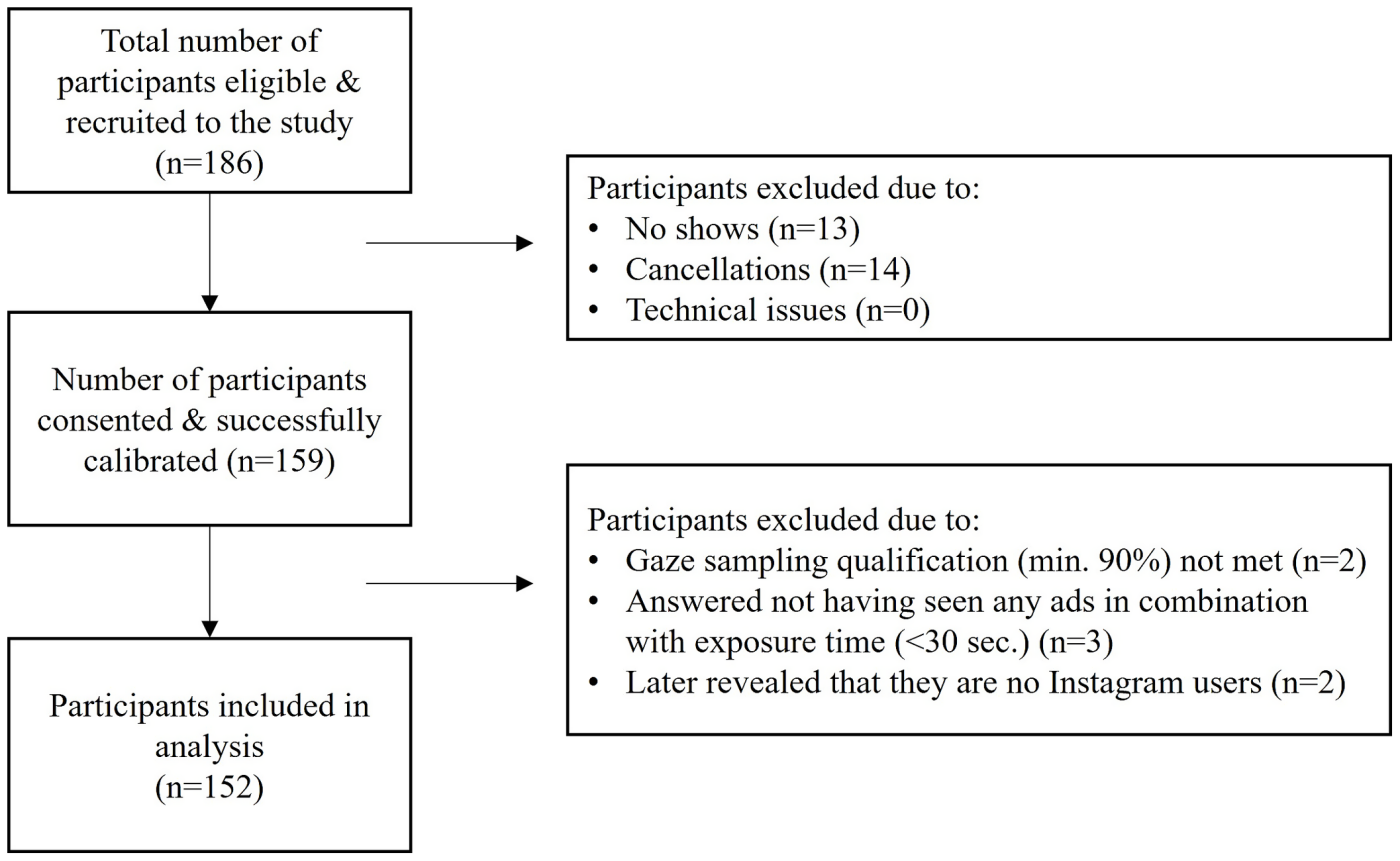
Participant recruitment and inclusion process.

Figure 1 provides a visual breakdown of the participant recruitment process, detailing the inclusion criteria and data-cleaning steps. Of the 186 initially recruited participants, after screening based on the exclusion criteria (e.g., not Instagram users, eye tracker calibration failures, and missing data), the final sample consisted of 152 participants (46.1% female and 53.9% male participants; mean age = 25.10 years, SD = 6.24), reflecting a broad representation of young adult social media users. This distribution aligns with Instagram’s primary user demographic in Germany (NapoleonCat, 2025). This sample size exceeds typical thresholds in eye-tracking research, where 10–30 participants are often sufficieant under tightly controlled conditions (Bojko, 2013; Holmqvist et al., 2012). Comparable recent studies using within-subjects eye-tracking designs have drawn valid inferences from samples ranging between 49 and 82 participants (e.g., García-Carrión et al., 2025; Ronconi et al., 2025). Therefore, the findings of this study are representative of typical Instagram users and their engagement with social media advertising.

### 2.6 Apparatus and procedure

**Figure 2:**
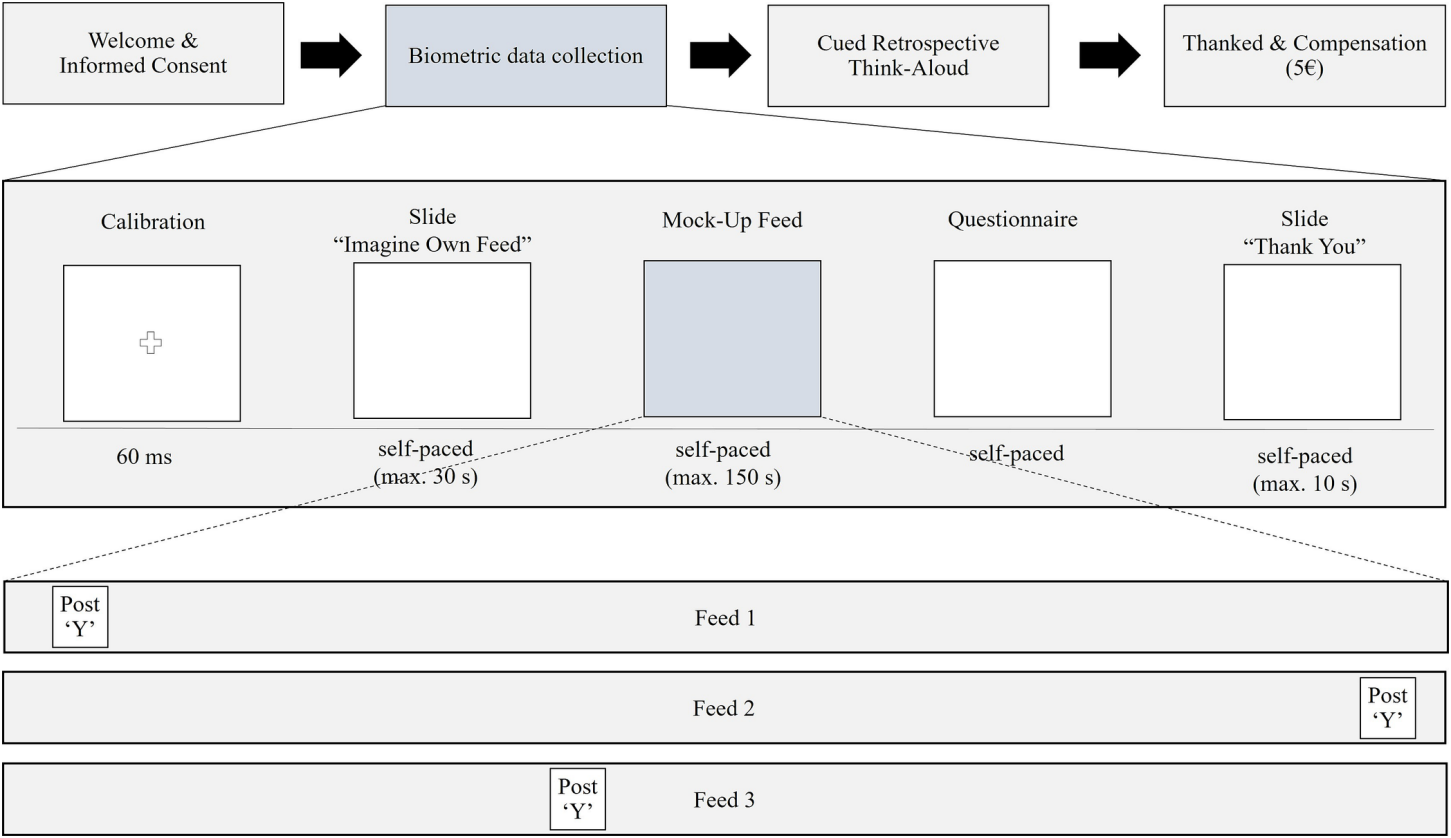
Study flow chart.

The study was conducted in a controlled laboratory environment at Ruhr West University of Applied Sciences, where participants completed the experiment individually. The entire session lasted approximately 30 min and followed a standardized protocol to ensure consistency across participants. Data collection was carried out using iMotions 9.3, an integrated biometric research platform that synchronized eye-tracking, galvanic skin response (GSR), and facial expression analysis. Although the GSR and facial expression data were collected for a separate study, these measures were unobtrusive and did not interfere with the eye-tracking procedure. The procedure was divided into the following five steps (illustrated in Figure 2).

Step 1: Upon arrival, participants were welcomed and received a brief overview of the study, which was framed as an investigation into Instagram browsing behavior. They were informed about the voluntary nature of their participation and provided written informed consent prior to their engagement. This study was approved by the ethics review board of the University of Twente (protocol number: 230564).

Step 2: Before beginning the experimental task, the participants were seated 65 cm from a 24-inch monitor with a resolution of 1920 × 1080 pixels. The eye-tracking system utilized was the Smart Eye AI-X infrared eye tracker, operating at 60 Hz with an accuracy of approximately 0.5° visual angle. Participants underwent a 9-point fixation calibration to optimize eye-tracking accuracy. Calibration ensured that gaze data were recorded reliably, minimizing drift and measurement errors. Once calibration was complete, the participants proceeded to the free-scrolling task.

Step 3: Before starting the browsing task, the participants were presented with an instructional slide containing a brief text introduction. This slide instructed them to imagine that the Instagram feed presented to them was their personal feed, containing posts from friends, influencers, and brands they might follow. They were asked to scroll and interact naturally, just as they would in their typical Instagram usage, including liking posts and swiping through multi-image carousels. The participants were then given access to a mock-up Instagram feed designed to replicate a naturalistic social media experience. They were given the freedom to scroll at their own pace, with a maximum browsing duration of 1 min 50 s.

Step 4: Following the browsing session, the participants completed a CRTA interview, lasting approximately 15 min. Participants were shown a replay of their own gaze patterns overlaid on the Instagram feed. They were prompted to explain their attention allocation, engagement decisions, and reasons for fixating on or skipping specific AOIs. The interviewer probed for additional insights, particularly regarding ad recognition, processing strategies, and avoidance behaviors.

Step 5: Upon completion of the study, participants were thanked for their participation and compensated with €5 in cash. The compensation amount was determined based on common research practices for studies of similar length and complexity. The monetary compensation was set at a level that acknowledged the participants’ time and effort, without being so high as to induce participation solely for financial reasons.

### 2.7 Measures and data preparation

#### 2.7.1 Quantitative eye-tracking data (stages 1 − 3a)

**Figure 3:**
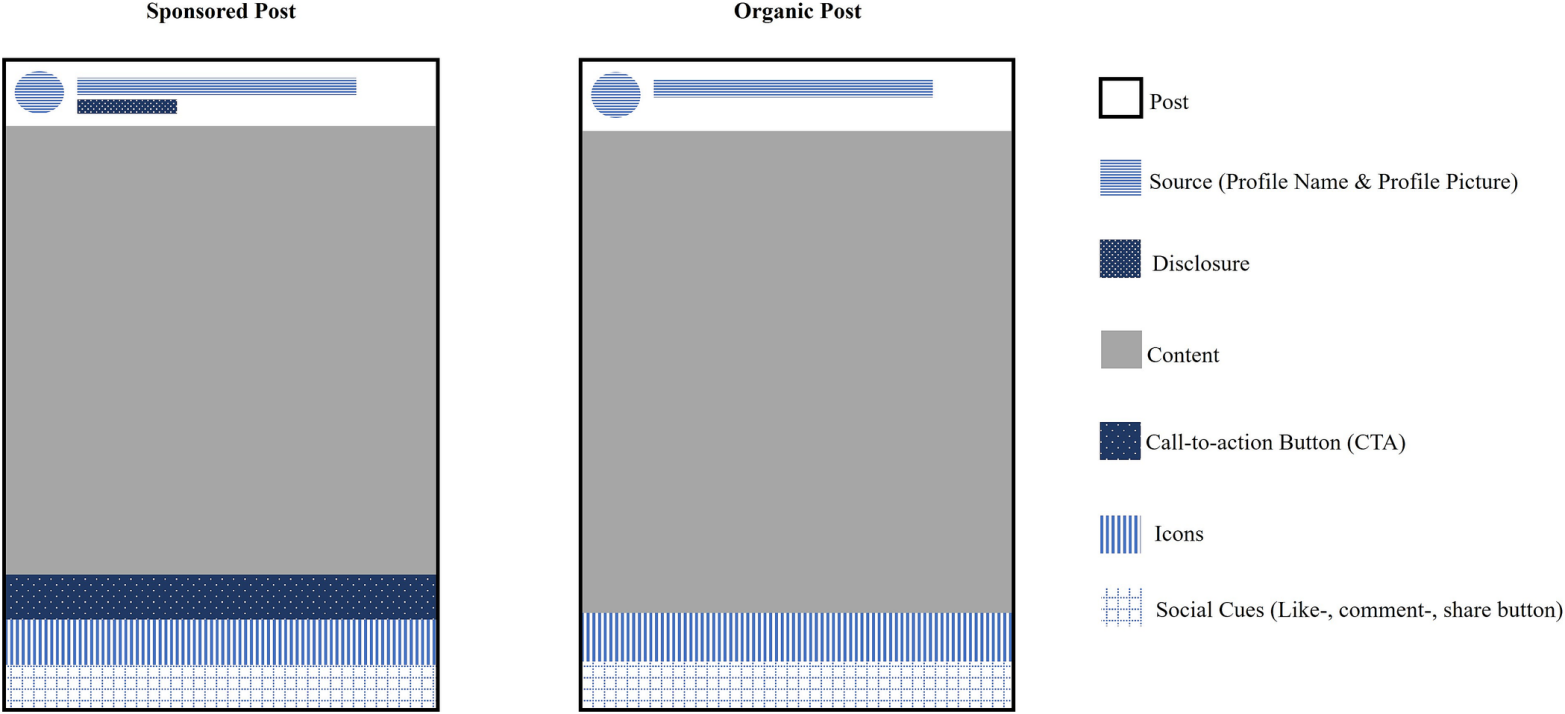
Visual representation of defined areas of interest (AOIs) in sponsored and organic instagram posts.

To capture how users visually engage with Instagram posts, distinct AOIs were defined for both sponsored and organic content. These AOIs reflect the structural components of a typical Instagram post, encompassing both advertising-specific elements (e.g., disclosures, CTA buttons) and universal post features (e.g., content area, source information). Eye-tracking metrics provide an objective assessment of attention allocation, revealing which post elements attract users’ gaze and for how long. The selection of AOIs was grounded in visual hierarchy principles (Henderson and Hayes, 2018), attention-based advertising research (Wedel and Pieters, 2008), and user engagement frameworks (Cialdini, 2001) (see Table 2). The following AOIs were drawn, illustrated in Figure 3.

**Table 2:** Operationalization of Instagram post elements as areas of interest (AOIs) with theoretical rationale.

AOI	Description and function	Justification for inclusion
AOI 1: Content Area (e.g., the Image)	Main visual content of the post.	The largest and most salient area, expected to receive highest dwell time (Pieters and Wedel, 2004). Determines whether users engage before recognizing sponsorship.
AOI 2: Icons (Likes, Comments, Share Icons)	Engagement buttons (likes, shares, etc.).	Represents interactive engagement (social proof theory, Cialdini, 2001).
AOI 3: Source (Profile Name and Profile Picture)	Identifies the account posting the content.	Users rely on profile cues to assess source credibility (trust transfer theory; Stewart, 2003). Essential for schema-based recognition of brand accounts vs. influencers.
AOI 4: Social Cues	Like counts, comment counts, and engagement metrics.	Social proof theory (Cialdini, 2001) suggests that users rely on peer engagement for credibility assessment. Prior work shows ads receive lower social cue engagement (Maslowska et al., 2021).
AOI 5: Call-to-Action (CTA) Button *(Only in Sponsored Posts)*	Clickable button (e.g., “shop now”).	Functional element prompting further interaction. If fixated early, it may act as an advertising trigger, leading to ad avoidance (Windels et al., 2018).
AOI 6: Disclosure Label (“Sponsored”) *(Only in Sponsored Posts)*	Explicit sponsorship cue.	Critical for ad recognition (Boerman and Müller, 2022). Short fixation times indicate schema-driven recognition, while longer fixations suggest user uncertainty.
AOI 7: Overall Post Boundary *(Only for Comparative Analysis)*	Entire post boundary, encompassing all AOIs.	Used to measure total dwell time per post (sponsored vs. organic), indicating overall engagement levels.

The selection of eye-tracking metrics (fixation count, dwell time, and TTFF) was driven by theoretical and empirical considerations regarding visual attention in digital environments, particularly in the context of advertising recognition and avoidance. Each metric captures distinct aspects of how users allocate attention to different elements within an Instagram feed. Fixation count refers to the number of times a participant’s gaze stops at a specific AOI. Higher fixation counts typically indicate increased cognitive processing and visual engagement (Holmqvist et al., 2012). This metric is essential for understanding whether users actively engage with ad disclosures and CTA buttons or if these elements are ignored due to learned avoidance (Orquin and Wedel, 2020). Given this study’s focus on advertising recognition and avoidance mechanisms, fixation count serves as an objective indicator of how often users look at key ad elements. Dwell time (ms) is the total duration (in milliseconds) that participants spend fixating within an AOI. It provides a more nuanced understanding of cognitive effort and engagement levels, particularly in distinguishing between automatic ad avoidance and deliberate engagement with ad elements (Maslowska et al., 2021). A low dwell time suggests that the ad cue was seen but dismissed quickly (schema-based recognition). A higher dwell time may indicate deeper processing of content, aligning with DPTs of persuasion (Chaiken et al., 1989; Kahneman, 2011; Petty and Cacioppo, 1986). TTFF measures the time elapsed before a participant first fixates on a given AOI. This metric is particularly important in determining whether disclosures or CTA buttons function as “ad flags” that may immediately trigger ad avoidance. If disclosure are among the first elements fixated upon, and the subsequent dwell time decreases, this provides evidence for heuristic-driven ad recognition. Conversely, a delayed TTFF suggests that users do not immediately perceive the post as sponsored content, supporting the argument that native advertising remains partially camouflaged.

Eye-tracking data are inherently complex and can exhibit a wide range of distributions owing to the variability in human visual attention and cognitive processing (Li et al., 2020; Visweswaran et al., 2021). To account for this variability in participants viewing behaviors and scrolling speeds, the data were examined for skewness and kurtosis. Initial analysis indicated high skewness (>3) in 12.5% (M32) and 18.75% (M27) of cases, and excessive kurtosis (>3) in 67.5% (M32) and 75% (M27) of cases, suggesting non-normality. Given that parametric statistical tests assume normal distributions, data transformation was applied (D. K. Lee, 2020; Nahm, 2016). Both log transformation and square root transformation were evaluated as potential solutions (Blascheck et al., 2017; Maddox et al., 2018). While square root transformation improved normality to some extent, it did not sufficiently reduce skewness and kurtosis across all variables (e.g., M32_sqrt: 18.13% of cases still exceeded kurtosis >3; M27_sqrt: 5.63% still exceeded skewness >3). In contrast, log transformation eliminated all skewness and substantially reduced kurtosis (100% reduction for M32 and 83.33% for M27). Based on these results, log transformation was selected as the most effective normalization technique. These transformations ensured the appropriateness of subsequent statistical analyses, aligning with best practices in behavioral and eye-tracking research (Gidlöf et al., 2013; Hu et al., 2024).

#### 2.7.2 Qualitative data from CRTA interviews (stage 3b)

While the eye-tracking data from Stages 1 − 3a demonstrate how participants allocate attention to (or away from) ad disclosures and CTA buttons, they do not fully explain why users engage with certain elements while ignoring others. To gain deeper insights into the cognitive and affective processes guiding user interactions with sponsored content, CRTA interviews were conducted immediately after the eye-tracking experiment (Bruun et al., 2021). Participants reviewed their own gaze patterns and described their thought processes as they engaged with different content types. For the qualitative analysis, a hybrid thematic coding approach (deductive-inductive) was applied (Bihu, 2024; Fereday and Muir-Cochrane, 2006). While deductive coding was grounded in established theories of persuasion knowledge (PK), heuristic ad avoidance, and visual engagement models, the inductive approach ensured that emerging, data-driven insights—such as esthetic expectations, thematic fit, and engagement rationalization—were systematically integrated. The deductive framework enabled alignment with prior findings, ensuring that ad recognition strategies (e.g., disclosures) and avoidance mechanisms (e.g., habitual scrolling, disengagement upon recognizing persuasion) were consistently assessed. Inductive coding allowed for data-driven expansion, capturing novel behavioral patterns and emerging psychological mechanisms. Reliability was reinforced through multiple coders, enhancing analytical robustness. The Holsti index (Holsti, 1969) indicated substantial agreement (99.2%) (Landis and Koch, 1977), ensuring coding consistency and minimizing subjective bias (Atlas.ti, 2019).

## 3 Results

### 3.1 Overall visual attention differences (sponsored vs. organic)

To examine the differences in visual attention between sponsored and organic posts, we conducted a repeated-measures ANOVA (within-subject factor: sponsorship status) on log-transformed dwell times (AOI 7, overall post boundary). The analysis revealed a significant main effect of post type (sponsored vs. organic) on total dwell time, *F* (1,151) = 8.338, *p* = 0.004, η^2^ = 0.052, indicating a statistically significant but small-to-moderate effect size. This is consistent with real-world behavioral data, where differences in attention tend to be subtle but meaningful. Descriptive statistics showed that the mean dwell time on organic posts (*M* = 7,781 ms, SD = 2744.86) was higher than that on sponsored posts (*M* = 7,531 ms, SD = 3396.99). The mean difference in the log-transformed dwell time (0.0685) corresponds to an approximately 6.7% reduction in attention toward sponsored posts when back-transformed to the original scale. This result suggests that users, on average, allocate less total viewing time to sponsored content. The partial eta-squared value (η^2^ = 0.052) suggests that while the effect is statistically significant, its practical impact is moderate. A separate repeated-measures ANOVA for fixation count (log-transformed AOI 7) showed a highly significant effect of post type [*F* (1,151) = 102.503, *p* < 0.001, η^2^ = 0.404], indicating that 40.4% of variance in fixation count can be attributed to whether a post is sponsored or organic. Descriptive statistics confirmed that, on average, organic posts received 25% more fixations than sponsored posts (M_organic = 6.52, SD = 4.31; M_sponsored = 5.00, SD = 3.34). This result suggests that, while users may still spend time on sponsored posts, they interact with organic posts in a more fragmented and visually engaged manner. While differences in dwell time were numerically small on the log scale, back-transformation demonstrated a meaningful effect in real-time engagement: participants viewed organic posts approximately 250 ms longer than sponsored posts.

### 3.2 AOI-level visual attention differences

To better understand which individual post elements (previously defined as AOIs) contribute to the overall differences in attention between sponsored and organic content, a repeated-measures ANOVA was conducted. This analysis examined visual attention measures (dwell time and fixation count) across the distinct AOIs of Instagram posts while accounting for sponsorship status (sponsored vs. organic).

First, we conducted a 2 × 5 repeated-measures ANOVA (post type: sponsored vs. organic; AOIs 1–4 and 7: post content image, icons, source (including profile name and profile picture), social cues, and the overall post boundary) to examine how sponsorship affects attention allocation across multiple AOIs simultaneously. The results revealed a strong main effect of AOI on visual attention [*F* (4,132) = 105.78, *p* < 0.001, η^2^ = 0.762, large], confirming that different elements of the post attract significantly different levels of attention. A small but significant main effect of sponsorship on visual attention was noted [*F* (1,135) = 5.83, *p* = 0.017, η^2^ = 0.041, small], indicating that, on average, users allocate slightly different attention depending on whether the post is sponsored or organic. We also found a strong AOI × sponsorship interaction effect [*F* (4,132) = 93.25, *p* < 0.001, η^2^ = 0.739, large], suggesting that the effect of sponsorship is not uniform across all AOIs, but rather specific to certain elements of the post. Although sponsorship had a small effect when examined at the AOI level, prior analysis focusing only on the overall post-level (AOI 7) showed a more pronounced difference in dwell time between sponsored and organic posts [*F* (1,151) = 8.338, *p* = 0.004, η^2^ = 0.052, small-to-moderate]. This discrepancy suggests that sponsorship influences post-level engagement more than it does attention to individual elements. When examining AOIs separately, some (e.g., social cues, icons) exhibit strong sponsorship effects, whereas others (e.g., source) do not, diluting the sponsorship effect in the AOI-level analysis. This outcome suggests that users engage with the post as a whole differently from how they engage with its individual elements. The stronger post-level sponsorship effect may indicate an initial filtering process, where users categorize content based on ad recognition cues and adjust engagement accordingly. Including AOI 7 (overall post) in the analysis allowed us to compare the overall post with its individual components (AOIs 1–4), thereby showing how attention is distributed between the entire post versus its sub-elements; however, on the downside, we cannot isolate whether the effects are driven by the whole post or specific AOIs with these results. To differentiate post-level versus AOI-level effects more clearly, that is, to better understand which AOIs contribute to the observed differences, separate repeated-measures ANOVAs were conducted for each AOI (AOIs 1–4) and each visual attention metric (dwell time, fixation count). The results of these inferential tests are summarized in Table 3, which presents the statistical differences in visual attention across AOIs as a function of post type (sponsored vs. organic).

**Table 3:** Results of repeated-measures ANOVAs comparing visual attention to Instagram post elements (AOIs) by sponsorship type.

				RM ANOVA						
	Wilks’λ	F (df1, df2)	*p*-value	η²	*M*	SD	*M*	SD	ΔM	SD
				(Effect size)	(Sponsored)	(Sponsored)	(Organic)	(Organic)		
AOI 7 (Overall)										
Fixation count (log transf.)	0.596	102,503 (1, 151)	0.000	0.404	1.460	0.042	1.747	0.038	0.28	0.028
Dwell time (log transf.)	0.948	8,338 (1, 151)	0.004	0.052	8.831	0.036	8.900	0.028	0.069	0.024
AOI 1 (Content)										
Fixation count (log transf.)	0.636	86,365 (1, 151)	0.000	0.364	1.177	0.039	1.437	0.034	0.260	0.028
Dwell time (log transf.)	0.955	7,133 (1, 151)	0.008	0.045	8.674	0.040	8.738	0.032	0.064	0.024
AOI 2 (Icons)										
Fixation count (log transf.)	0.784	38,071 (1, 138)	0.000	0.216	0.241	0.030	0.470	0.027	0.229	0.037
Dwell time (log transf.)	0.902	15,039 (1, 138)	0.000	0.098	7.950	0.095	8.279	0.053	0.329	0.085
AOI 3 (Source; Profile Picture and Name)										
Fixation count (log transf.)	0.889	18,743 (1, 150)	0.000	0.111	0.237	0.020	0.348	0.023	0.111	0.026
Dwell time (log transf.)	0.999	0,216 (1, 150)	0.642	0.001	8.050	0.072	8.081	0.058	0.031	0.066
AOI 4 (Social Cues)										
Fixation count (log transf.)	0.767	44,553 (1, 147)	0.000	0.233	0.450	0.036	0.676	0.032	0.226	0.034
Dwell time (log transf.)	0.982	2,754 (1, 147)	0.099	0.018	8.319	0.069	8.413	0.044	0.094	0.056

Results show that for AOI 1 (content area), the fixation count was significantly higher for organic posts than for sponsored posts [*F* (1,151) = 38.65, *p* < 0.001, η^2^ = 0.364, large], indicating that users scanned organic content more often. However, dwell time differences were less pronounced but still significant [*F* (1,151) = 8.177, *p* = 0.004, η^2^ = 0.045, small], suggesting that while organic content attracted more fixations, the overall viewing duration was only slightly longer. However, for AOI 2 (icons, engagement buttons), both fixation count [*F* (1,151) = 30.71, *p* < 0.001, η^2^ = 0.216, moderate-to-large] and dwell time [*F* (1,151) = 9.38, *p* = 0.003, η^2^ = 0.098, moderate] were higher for organic content, suggesting that users paid more attention to interactive elements when content was not explicitly labeled as advertising. Regarding AOI 3 (source: profile name and profile picture), results show that while fixation count was significantly higher for organic content [*F* (1,151) = 11.34, *p* = 0.001, η^2^ = 0.111, moderate], dwell time did not differ significantly [*F* (1,151) = 0.21, *p* = 0.642, η^2^ = 0.001, negligible]. This result suggests that users frequently checked the source of organic posts but did not necessarily spend more time evaluating it. As for AOI 4 (social cues, likes and comments), a large effect was observed for fixation count [*F* (1,151) = 44.55, *p* < 0.001, η^2^ = 0.233], meaning that users actively scanned social cues more often in organic posts. However, dwell time differences were not significant [*F* (1,151) = 2.754, *p* = 0.099, η^2^ = 0.018, negligible], suggesting that users glanced at social metrics frequently but processed them quickly. For additional context, the back-transformed descriptive statistics for all AOIs and both attention measures are reported in Table 4, providing interpretable mean values for sponsored and organic content.

**Table 4:** Means and standard deviations of back-transformed visual attention measures (fixation count and dwell time) for sponsored and organic posts across post elements (AOIs).

	Sponsored					Organic				
	*n*	Min.	Max.	*M*	SD	*n*	Min.	Max.	*M*	SD
AOI 7 (Overall)										
Fixation count (exp. back-transf.)	152	1.75	21.33	5.00	3.34	152	2.86	36	6.52	4.31
Dwell time (exp. back-transf.)	152	1302.22	19276.40	6571.87	3293.00	152	1921.53	17038.47	6736.72	2742.26
AOI 1 (Content)										
Fixation count (exp. back-transf.)	152	1.38	13.00	3.70	2.33	152	2.05	23.14	4.69	2.82
Dwell time (exp. back-transf.)	139	99.97	27540.73	4723.98	4890.90	152	633.35	18278.95	4743.10	2983.34
AOI 2 (Icons)										
Fixation count (exp. back-transf.)	139	1.00	4.50	1.37	0.65	152	1.00	4.33	1.68	0.62
Dwell time (exp. back-transf.)	151	166.67	18934.70	4452.87	3867.49	152	275.03	14706.59	4065.15	2837.00
AOI 3 (Source; Profile Picture and Name)										
Fixation count (exp. back-transf.)	151	1.00	3.00	1.31	0.37	152	1.00	5.14	1.48	0.53
Dwell time (exp. back-transf.)	148	133.35	29902.13	5631.75	4772.20	152	1035.52	13742.13	5142.99	2698.65
AOI 4 (Social Cues)										
Fixation count (exp. back-transf.)	148	1.00	6.50	1.74	0.94	152	1.00	8.25	2.14	1.10
Dwell time (exp. back-transf.)	148	133.35	29902.13	5631.75	4772.20	152	1035.52	13742.13	5142.99	2698.65

### 3.3 Disclosures and CTA buttons as ad “flags”

Next, we aimed to determine whether early fixation (TTFF) on disclosures (AOI 6) and CTA buttons (AOI 5) functions as a perceptual “flag,” prompting users to disengage more quickly from sponsored content. According to the PKM (Friestad and Wright, 1994), schema theory (Bartlett, 1932), and the LC4MP (Lang, 2017), users who recognize ad signals early are expected to activate cognitive resistance mechanisms, leading to a reduction in the overall dwell time on the main content of the post (AOI 1). The eye-mind hypothesis (Just and Carpenter, 1980) suggests that visual fixations reflect cognitive processing, allowing us to examine whether early attention to disclosures or CTA buttons alters subsequent engagement patterns.

To assess whether early fixation on disclosures (AOI 6) or CTA buttons (AOI 5) functions as an “ad flag” effect, resulting in a change in dwell time, we categorized participants based on their first fixation location. Specifically, we examined (1) whether the disclosure (AOI 6) was the first fixation (AOI 6_First); (2) whether the CTA button (AOI 5) was the first fixation (AOI 5_First); (3) whether participants first fixated on the disclosure, then the CTA button (AOI 6_AOI 5); and (4) whether participants first fixated on the CTA button, then the disclosure (AOI 5_AOI 6). Therefore, 31 separate univariate ANOVAs (UNIANOVAs) were conducted—one for each dependent variable (i.e., dwell time on a specific AOI for a specific post)—each including the four predictors (AOI 5_First, AOI 6_First, AOI 6_AOI 5, and AOI 5_AOI 6). Because each UNIANOVA tested four predictors, a total of 128 individual F-tests were performed (some were missing due to data constraints).

The proportion of significant and marginally significant findings relative to the total number of tests is summarized in Tables 5, 6, provides a breakdown by effect type. Given that this manuscript focuses on generalizable attention patterns rather than post-specific effects, we do not list the results for individual posts. Instead, we report the frequency of significant effects as proportions of the total analyses conducted, providing a broader perspective on how early ad recognition influences subsequent visual engagement across multiple post elements. The results indicate that 24 out of 31 tests (77.4%) yielded statistically significant effects (*p* < 0.05), while an additional 7 tests (22.6%) were marginally significant (*p* = 0.055–0.099). Breaking these results down by effect type, early fixation on the CTA button (AOI 5_First) produced the highest number of significant effects (13 out of 24, 54.2%), whereas early fixation on the disclosure (AOI 6_First) followed closely, yielding 10 significant effects (41.7%). Sequential fixation patterns, such as first fixating on the disclosure (AOI 6) followed by the CTA button (AOI 5) (AOI 6_AOI 5) as an effect type was not significant, while vice versa (AOI 5_AOI 6), showed one significance test.

**Table 5:** Effects of early fixations on ad flags (CTA buttons and disclosures) on dwell time.

Effect type	Total number	% of total tests
Significant (*p* < 0.05)	24	77%
Marginally significant (0.055–0.099)	7	23%
Total	31	100%

**Table 6:** Breakdown of effects by first fixation type.

Effect type	Significant (*p* < 0.05)	% of Sig. Effects	Marginal (0.055–0.099)	% of Marginal effects
AOI 5_First	13	54%	2	29%
AOI 6_First	10	42%	2	29%
AOI 6_AOI 5	0	0%	3	43%
AOI 5_AOI 6	1	4%	0	0%

To further investigate the direction of these effects, we compared the mean dwell times between conditions where early fixation on the CTA button (AOI 5) or the disclosure (AOI 6) was present versus absent. The results indicate that in 25 cases (80.6%), the significant effect resulted in a decrease in dwell time, meaning that early ad recognition led to faster disengagement from the post’s main content (AOI 1). In six cases (19.4%), the effect resulted in an increase in dwell time, suggesting prolonged engagement with certain elements of the post. Notably, four of the six increases in dwell time (66.7%) occurred for the source information (AOI 3, profile name and profile picture). This systematic trend aligns with the findings from our repeated-measures ANOVA results, which showed a significant difference in dwell time—but not fixation count—between the sponsored and organic content for AOI 3. This finding suggests that certain cognitive or perceptual processes associated with evaluating the source of a post warrant further investigation. Users may spend more time engaging with profile information when experiencing uncertainty about sponsorship status, particularly if the disclosure cues fail to provide immediate clarity.

### 3.4 Reasoning of attention allocation: qualitative analysis (CRTA interviews)

While the eye-tracking data from Stage 3a demonstrate how participants allocate attention to (or away from) ad disclosures and CTA buttons, they do not fully explain why users attend to or ignore these “ad flags.” To gain a deeper understanding of the participants’ subjective experiences and motivations, we conducted CRTA interviews. For qualitative analysis of the CRTA interview data, thematic coding was performed using a deductive-inductive approach.

The findings reveal that ad recognition is a multilayered cognitive process, shaped by visual, textual, and structural cues. Participants relied on multiple design elements to identify advertising, with variations in the immediacy and accuracy of their recognition. This is shaped by esthetics, thematic relevance, cognitive load, and long-term exposure effects. While some users exhibited strong predispositions toward avoiding sponsored content, others engaged when certain psychological triggers were activated.

The data highlight that the design elements of social media posts play a fundamental role in recognition. A key finding from the CRTA analysis challenges prior literature, which has largely focused on disclosures as the primary ad recognition cue (Wojdynski and Evans, 2020). While disclosures remained an important indicator (95 mentions), participants also frequently referenced CTA buttons as a decisive factor in recognizing content as sponsored (77 mentions). Additionally, participants noted that profile information, such as verification badges (27 mentions) or brand logos as profile pictures (81 mentions), influenced their perception of a post’s authenticity and its classification as organic or sponsored content. Additionally, participants reported that the explicitness of the content image itself played a pivotal role in recognition. The degree to which the product was clearly displayed within the post—whether it was immediately recognizable or embedded more subtly—determined how quickly users categorized the post as an advertisement (178 mentions). Participants repeatedly emphasized the importance of clear and immediately recognizable content (59 mentions), highlighting the role of visual saliency: “*The way the product was presented made it obvious it was an ad, even before I noticed anything else”* (Participant 61); this exemplifies System 1 processing, wherein recognition occurs effortlessly and instantaneously based on familiar visual and structural cues.

However, despite the recognition, this did not automatically lead to disengagement. Another recurring theme in participant responses was regarding esthetic quality, which plays a pivotal role in engagement, yet its influence is paradoxical: high perceived esthetic value enhanced engagement but also triggered skepticism by signaling professionalization, which users equate with advertising: “*I would even like an ad when it looks like they put a lot of effort into it in terms of esthetics*” (Participant 38). However, when visuals appeared overly polished or “too professional,” some participants expressed distrust and disengagement. This finding suggests that esthetic expectations serve as a heuristic cue in ad recognition—highly stylized visuals increase the likelihood of users classifying a post as a sponsored ad, whether correctly or incorrectly: “*If it looks too perfect, I just assume it’s an ad and ignore it*” (Participant 17).

Beyond esthetics, the perceived thematic fit between the ad and a user’s interests emerged as a dominant determinant of engagement. Users did not outright reject ads but rather filtered them through a personal relevance lens: “*Yes, another ad, but somehow that appealed to me. Well, I thought it was cool. […] I have to say, I did not even realize that it was advertising, so I probably did just like it because it I am interested in this kind of topic*” (Participant 66). Conversely, when an ad lacked thematic fit, even high production value did not prevent disengagement: “*The picture was nice, but I wasn’t interested in the product, so I moved on*” (Participant 68); another participant stated: *“If I like something, I do not care if it’s an ad—I want to see more of it”* (Participant 74). However, some participants also mentioned the following: *“Sometimes I like ads on purpose so I get more offers in my feed”* (Participant 61) and *“I do not mind ads if they are about things I actually like or if they look really good”* (Participant 122). Interestingly, some users even viewed sponsored posts as a strategic tool to optimize their feed preferences, expecting better-targeted promotions, exclusive deals, or promo codes. This supports the notion that engagement with advertising is not purely passive but can be an active and deliberate behavior.

The impact of repeated ad exposure revealed two divergent psychological effects: (1) the mere-exposure effect (Bornstein and Craver-Lemley, 2022) and (2) general ad fatigue (Elsen et al., 2024). In line with the first effect, some participants acknowledged that despite initial avoidance, repeated exposure to the same brand gradually built familiarity and trust, increasing the likelihood of future engagement. At the same time, others reported irritation upon seeing the same ad too frequently, leading to deliberate avoidance, representing the second effect: “*At first, I ignored the brand, but after seeing it multiple times, I started to recognize and trust it*” (Participant 69); “*If I see the same ad too often, I just get annoyed and block it*” (Participant 74). This dual effect of repeated exposure suggests that frequency-capping strategies should be optimized—too little exposure fails to build recognition, whereas excessive repetition triggers disengagement.

## 4 Discussion

This study offers empirical insights into the mechanisms underlying consumer attention allocation in social media feeds, advancing our understanding of how and why users engage with (or disengage from) sponsored and organic content. The findings underscore an ongoing theoretical debate: Do native ad disclosures function as effective transparency cues, prompting consumer awareness, or do they serve as ad avoidance triggers, reinforcing disengagement? By integrating elements of banner blindness, dual-process models, PK, and schema theory, we reveal that consumer interactions with social media posts are governed by a complex interplay of cognitive filters, attentional limits, and automated disengagement mechanisms. The implication is that users do not necessarily ignore ads outright but employ a rapid cognitive filter to determine whether a post is worth processing further.

Regarding the overall visual attention differences based on post type (sponsored vs. organic), our findings align with previous research on advertising avoidance behavior, where subtle but consistent patterns indicate reduced engagement with overtly commercial content (Boerman et al., 2017; Wojdynski and Evans, 2016). Furthermore, organic posts elicited significantly more fixations, suggesting greater cognitive processing and engagement with non-sponsored content. These findings provide empirical support for banner blindness theory (Benway, 1998), extending it to native ad formats. Despite the seamless integration of sponsored posts, participants exhibited learned avoidance by allocating significantly less attention to them. This aligns with the PKM (Friestad and Wright, 1994), suggesting that even subtle disclosures trigger coping strategies, leading users to ignore or minimize their interaction with sponsored content. Moreover, the increased TTFF on sponsored posts suggests a hesitancy effect, aligning with DPTs. Participants may have engaged in System 1 (heuristic) processing, where subtle cues such as a “sponsored” label prompt immediate disengagement before deeper content evaluation can occur. Despite their seamless design, native ads still suffer from avoidance, with reduced attention metrics across all measures compared with organic posts. This finding suggests that users rely on quick heuristics to detect and ignore persuasive content, reinforcing an updated form of banner blindness in social media feeds.

The results of the AOI-level visual attention differences align well with the covered advertising recognition and effects model (CARE) (Wojdynski and Evans, 2020), which suggests that schema-driven categorization (top-down processing) enables users to recognize branded content rapidly. By contrast, bottom-up recognition processes, such as the presence of CTA buttons or engagement cues (likes/comments), trigger prolonged attention as users subconsciously verify a post’s intent. These findings highlight how users selectively allocate cognitive resources, prioritizing ad-relevant features only when their recognition schema is not already activated.

This is further reinforced by the strong fixation count effects in AOI 2 (icons) and AOI 4 (social cues) (see Table 4), which indicate that users scan these areas more actively in organic posts but do not necessarily dwell longer, possibly because engagement elements are more salient in organic content. The lack of a significant dwell time difference in AOI 3 (source) despite more fixations suggests that users verify the source identity more often in organic posts but require minimal processing time. By contrast, dwell time differences in AOI 1 (content image) were smaller than fixation count effects, suggesting that users frequently glance at organic images but spend comparable durations engaging with content across conditions.

The higher fixation count for social cues (AOI 4) in organic posts indicates that users are frequently “checking” these, perhaps to assess authenticity or peer validation. This behavior is consistent with bottom-up recognition mechanisms, wherein unexpected engagement metrics (e.g., more likes) may signal organic content, prompting further inspection. Yet, in sponsored posts, users may assume persuasive intent (top-down recognition), reducing their need to scan social cues for credibility (Wojdynski and Evans, 2020). However, the lack of a dwell time difference suggests that although fixations are more frequent, they are shorter in duration, implying superficial or habitual processing rather than in-depth evaluation. This pattern reflects a “scanning behavior”—quick glances to gather surface-level information. Indeed, Kahneman (2011) suggests that System 1 (automatic processing) likely governs social cue processing, especially in familiar environments such as Instagram.

This explains the frequent but brief fixations observed—users rely on automatic, fast assessments without extensive cognitive effort. System 2 (deliberate processing) seems to be activated when evaluating content credibility (AOI 3: source in terms of profile picture and profile name), explaining the significant dwell time differences found in this process. In light of the differences found in AOI 4 and AOI 3 regarding dwell time and fixation count, one may draw from social proof theory (Cialdini, 2001). In organic posts, the social cues (e.g., likes, comments) may carry greater informational value, leading to more frequent fixations as users seek validation cues. Meanwhile, in sponsored posts, users might expect promotional content, reducing the need to verify authenticity through social cues, hence involving fewer fixations.

This finding is further supported by the results regarding “ad flags,” showing that when disclosures or CTA buttons are fixated on early, dwell time on the entire post significantly decreases. These findings challenge the widely held assumption that disclosures (AOI 6) serve as the primary cue for distinguishing sponsored from organic content. Prior research has largely focused on disclosure labels as the critical trigger for ad recognition (Boerman and van Reijmersdal, 2016; Wojdynski and Evans, 2016), assuming that users primarily rely on these explicit markers to recognize advertising content. However, our results challenge this assumption by demonstrating that users actively attend to other ad cues—particularly CTA buttons (AOI 5). The CTA button (AOI 5), often labeled with action-oriented phrases such as “shop now” or “learn more,” emerged as the most frequently significant AOI, suggesting that users are particularly attuned to interactive prompts. This is likely because of affordance-based heuristics that guide user expectations toward a potential succeeding action (Wojdynski and Evans, 2020). Unlike disclosures (AOI 6), which require top-down cognitive processing to be interpreted as an advertising cue, CTA buttons function through automatic recognition of interface design patterns, signaling commercial intent through immediate visual affordances rather than textual processing. The prominence of AOI 5 as a significant predictor aligns with existing research on digital ad engagement, which highlights that users develop conditioned responses to interface elements that consistently signal commercial intent (Tsai et al., 2017). The relative strength of CTA button attention effects over disclosure effects suggests that sponsored content recognition may be driven more so by interactive prompts than by regulatory ad labels.

This result suggests that users engage in an initial assessment phase (System 1 processing; Kahneman, 2011), and upon recognizing an ad, activate an automated disengagement response. Thus, rather than purely “blending in,” native ads that contain prominent disclosures often “stand out” in a way that reinforces avoidance rather than engagement. This finding aligns with the PKM (Friestad and Wright, 1994), which claims that consumers apply learned strategies to detect and resist persuasive intent. Thus, the “disclosure dilemma” is not merely a question of visibility but of cognitive processing timing: If a disclosure is detected early in the visual sequence, it acts as an ad flag, reducing engagement. However, if it is detected only after initial interest has been established, users may continue engaging with the content before re-evaluating their stance. Finally, many participants struggled to recall their interactions, indicating that automatic scrolling behavior dominates much of their engagement with social media feeds; this points to a low cognitive effort environment, where only content that sparks immediate relevance or esthetic appreciation disrupts habitual scrolling behavior.

Nevertheless, the CRTA results highlight a nuanced processing of information and how that ad recognition does not automatically lead to disengagement. While this qualitative approach provided valuable insight into participants’ subjective experiences, it also carries the potential for retrospective rationalization, as participants may construct *post hoc* narratives when viewing their own eye-tracking data. We do not treat this as a confounding bias, but as an interpretive window into how users make sense of their attentional behavior in retrospect. First, the findings regarding ad recognition align with schema theory (Bartlett, 1932), which suggests that people rely on mental shortcuts to categorize information rapidly. When an image conforms too closely to their pre-existing schema of an advertisement (i.e., high-resolution images, curated compositions, product close-ups), users default to an avoidance response. Conversely, when an ad blends seamlessly with organic content (e.g., user-generated content styles, natural-looking images), users are more likely to engage, as their schema does not immediately flag it as promotional. Specifically, if the content matched the participants’ interests, they often engaged despite recognizing it as advertising, thereby aligning with DPTs of information processing (Chaiken et al., 1989; Kahneman, 2011; Petty and Cacioppo, 1986), which posit that deliberate cognitive engagement (System 2) can override automatic heuristic-based avoidance (System 1). Second, according to the LC4MP (Lang, 2017), users allocate finite cognitive resources to content deemed relevant, filtering out non-relevant stimuli. Thus, thematic alignment acts as a cognitive gatekeeper, dictating whether an ad will undergo deep processing or be dismissed at the heuristic level. Some even actively interacted with sponsored posts, liking or bookmarking them, particularly when they believed doing so could influence the algorithm to show them more relevant content.

Overall, these findings reinforce the importance of designing engaging, authentic, and relevant content that aligns with user expectations and cognitive processing tendencies. Sponsored content that mimics organic esthetics while maintaining transparency may enhance engagement while mitigating ad resistance. Future research should explore how sponsorship disclosure timing (early vs. delayed recognition) affects emotional and behavioral responses to advertising.

While our study provides strong empirical evidence for a filtering-based model of attention allocation, several limitations must be acknowledged. Although our study simulated Instagram feeds realistically, real-world distractions and multitasking behaviors may influence attention differently. Furthermore, prior research suggests that ad avoidance may be influenced by individual differences in PK. By segmenting users into high-PK versus low-PK groups, future research could further test whether high-PK users disengage significantly faster than low-PK users. Future research could also segment users based on their browsing habits to further refine the model. Moreover, not only browsing habits but also cultural and educational differences may influence responses to native advertising. While our sample was demographically aligned with Instagram’s primary user base—young adults aged 18–34—it was drawn from a university-affiliated recruitment process within Germany. This introduces limitations regarding the generalizability of our findings to older populations and to users from other cultural contexts. Although recruitment extended beyond the student population to include participants from the surrounding urban community, future studies should deliberately include a wider age range and a broader cross-section of educational backgrounds to improve external validity. In addition, native advertising norms, platform familiarity, and persuasion knowledge can differ across cultural settings, potentially shaping ad recognition and engagement in distinct ways. Cross-cultural replications could therefore test the boundary conditions of the disclosure dilemma model proposed here and clarify how cultural schemata modulate attention to persuasive cues in social media environments. Future research could expand extant knowledge by addressing the limitations of this study. Despite providing multiple themes (i.e., to simulate a broad user experience, posts spanned common Instagram themes), especially the findings of Stage 3b point to the importance of personalized content. Generally, users are familiar with particular sources (as they are aware of which brand profiles they might follow) and the activities of their friends and families, and they might not check the source area as often as in this study or interact differently, as the respondents of this study also mentioned that the newsfeed posts did not cover themes they usually find interesting. Further, instead of using a binary-coded fixation order, a continuous TTFF variable could determine whether a shorter TTFF to ad cues corresponds with stronger avoidance responses. Ad recognition may differ across social media platforms and websites. Testing whether platform design influences disclosure processing would extend the current findings.

This study contributes to theory by extending and challenging dominant assumptions in the literature on advertising recognition. While disclosures have traditionally been treated as the primary recognition mechanism, our findings show that interactive design elements—particularly CTA buttons—may serve as even more salient cues. This nuance deepens current models of persuasive processing and challenges the assumption that regulatory ad labels are the sole or primary driver of advertising recognition. From a practical standpoint, these results offer actionable insights for marketers designing native ads: although mimicking the visual style of organic content may delay detection and extend engagement, overt cues such as CTA buttons can rapidly trigger ad recognition and disengagement. From an ethical perspective, the findings raise questions about transparency and user autonomy: while “invisible” ads may be effective at circumventing user resistance in the short term, they risk eroding trust and undermining informed consent in digital media environments. Marketers should therefore weigh short-term engagement benefits against long-term relational costs. Designing for trust, not just click-through, may ultimately foster more sustainable and respectful user-brand relationships.

## Data Availability

The datasets presented in this article are not readily available because they are the basis for ongoing research. Data may be available from the corresponding author on reasonable request after the completion of the overarching research project (October 2026). Requests to access the datasets should be directed to maike.huebner@hs-ruhrwest.de.

## Supplementary material

Supplementary Table 1 Codebook for the CRTA.
